# Aneurysm of antecubital vein: an unusual complication of peripheral intravenous cannulation

**DOI:** 10.1186/1471-2482-7-9

**Published:** 2007-06-14

**Authors:** Debasish Debnath, Stuart Wallace, Evangelia Mylona, Fiona Myint

**Affiliations:** 1 Department of Surgery, North Middlesex University Hospital, London, N18 1QX, UK; 2 Department of Pathology, North Middlesex University Hospital, London, N18 1QX, UK

## Abstract

**Background:**

Intravenous cannulation is a very common procedure. Venous aneurysm secondary to peripheral intravenous cannulation is extremely rare. Moreover, venous aneurysm can mimic other conditions and may confuse the issue.

**Case presentation:**

We describe a case of a 45-year-old woman who was referred with the diagnosis of varicose vein of right arm. A history of intravenous cannulation at the same site was noted that raised suspicion. The swelling was compressible and turned out to be a venous aneurysm. The lesion was completely excised. Postoperative recovery was uneventful. Histology findings were in conformity with the preoperative diagnosis.

**Conclusion:**

Caution should be exercised in diagnosing varicose vein at a site that bears a history of intravenous cannulation. The case also raises an important issue regarding consent. Should patients undergoing peripheral intravenous cannulation be warned of this rare complication?

## Background

Venous aneurysm is an uncommon condition that can present in a myriad of fashions and cause diagnostic challenge. We describe a rare case of symptomatic venous aneurysm of the median antecubital vein secondary to intravenous cannulation, which was initially referred by the general practitioner as a varicose vein. The aneurysm was excised successfully without any complication. Clinical features, treatment and complications of venous aneurysms have been discussed. The case raises the issue of warning patients of a rare complication of a common procedure. A review of literature has also been performed. The optimum management of venous aneurysms remains unclear. There are three objectives of reporting this case- i) to make readers aware of this rare but potentially serious condition; ii) to highlight the need for optimising treatment of venous aneurysms, and iii) to raise a debate whether such a complication should be routinely mentioned while obtaining consent for establishing peripheral cannulation.

## Case presentation

A 45-year-old woman presented with a swelling in her right antecubital fossa. The only relevant past medical history was an episode of gastroenteritis and dehydration, for which she required hospital admission eight years previously. During that admission she had received intravenous fluid therapy via a cannula in the right antecubital fossa approximating to the same area as the swelling. She recalled developing the swelling few months following the episode. The swelling had steadily grown over time and become painful. There was no history suggestive of deep venous thrombosis of the right arm. There was no other significant past medical history.

**Figure 1:**
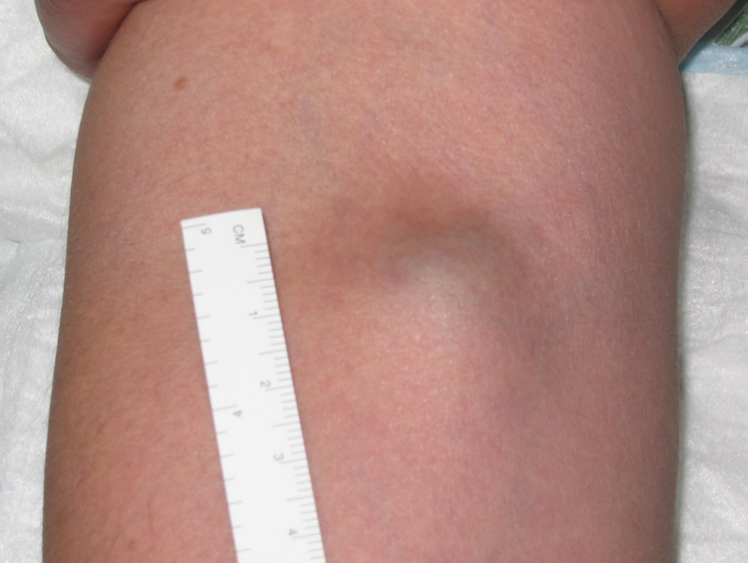
Soft compressible swelling on the right upper arm near antecubital fossa

General examination was unremarkable. Local examination showed a soft, non-tender, compressible lesion, 2 × 3 cm^2 ^in size, just distal to the right antecubital fossa [Figure 1]. There was no audible bruit. All the pulses in the arm were easily palpable. She was normotensive and there was no difference in blood pressure between the two arms. There was no neurological deficit.

Routine laboratory investigations, including coagulation profile, were within normal limits. She underwent a duplex scan that showed a venous aneurysm in relation to a large median cubital vein. The vein appeared competent. There was no evidence of ectasia, varicosity or thrombosis of any of the surrounding arm veins.

**Figure 2:**
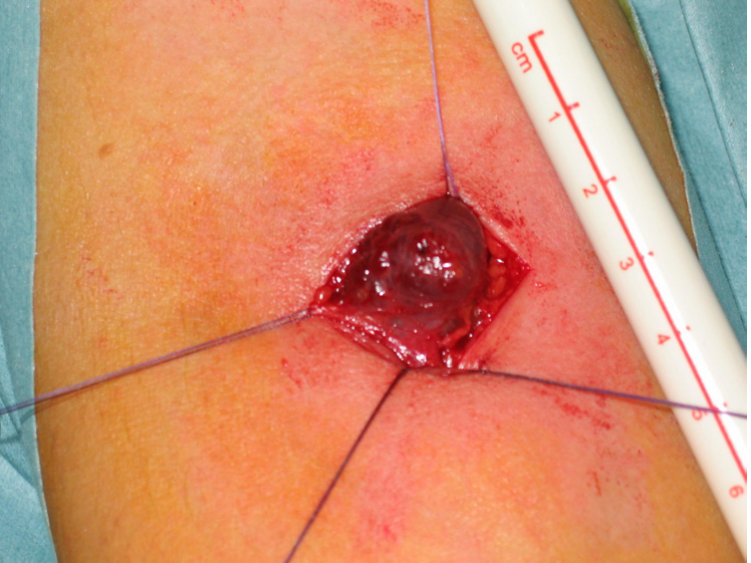
**Operative picture of the swelling**. Identification and ligation of the feeding vessels prior to excision are key operative steps.

The aneurysm was explored under general anaesthetic and was completely excised. The key operative steps involved identification and ligation of tributaries feeding the aneurysm, prior to excision of the sac [Figure 2]. She made an uncomplicated recovery with complete resolution of her symptoms.

**Figure 3:**
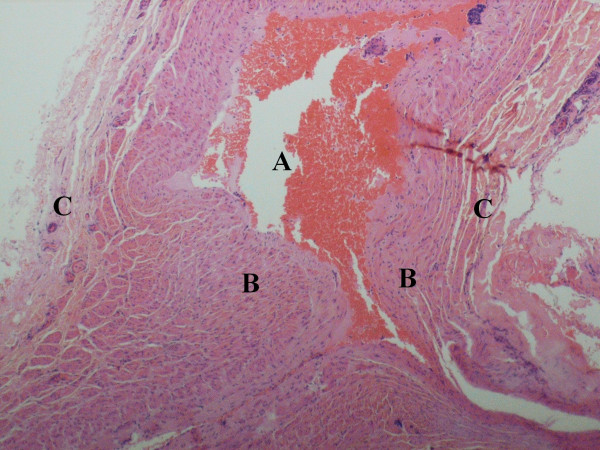
Microphotograph of the aneurysmal sac [H&E stain (10x0.30)] showing periphery of the blood vessel with congested lumen- [A] lumen [B] media [C] adventitia.

Histology of the aneurysmal sac showed periphery of the blood vessel with congested lumen and thickened media [Figure 3]. All three layers of the venous wall were preserved in the sac, which was suggestive of a true aneurysm. There was no element of endophlebosclerosis or endophlebohypertrophy.

## Discussion

Venous aneurysms are relatively rare. An unrestricted electronic search of the Medline database till February 2007 showed that one case of pseudoaneurysm of the antecubital vein had been described in the literature [1].

A venous aneurysm is defined as a dilatation of a localised segment of vein. Unfortunately there are no universally agreed size criteria to define a venous dilatation as an aneurysm. Aneurysm can be congenital or acquired, though the exact aetiology remains unclear. It has been suggested that aneurysmal dilatation is a response to increased haemodynamic pressure at a site of mural weakness [2]. However, aneurysms in the neck, where the pressure is low, cannot be explained by such theory [3]. It is possible that both congenital and acquired factors are involved. Lev and Saphir noted certain changes in gross and microscopic structure of the popliteal vein with age [4]. They described these changes as endophlebohypertrophy (intimal hypertrophy) and endophlebosclerosis (loss of smooth muscle and elastic tissue with replacement by connective tissue). These changes were seen at points of stress adjacent to arteries and entry points of tributaries. Schatz and Fine noted similar findings in venous aneurysms [5]. They considered endophlebohypertrophy and endophlebosclerosis as important factors in the development of venous aneurysms in a way similar to that of arteriosclerosis in the formation of arterial aneurysms.

Sites of aneurysm can be grouped as (i) central thoracic (e.g., superior vena cava); (ii) visceral (e.g., portal, superior mesenteric, splenic, renal); and (iii) cervical (e.g., jugular, facial, subclavian) and (iv) peripheral (e.g., cephalic, iliac, femoral, saphenous, popliteal) [6].

A venous aneurysm can present as a soft, compressible, subcutaneous mass that decompresses with elevation and enlarges with dependency and the Valsalva manoeuvre [6]. It can be detected as an incidental finding on an imaging study or diagnosed during investigations of a venous thromboembolic event. In the upper limb it is most likely to present as a soft tissue swelling that may or may not result in compressive symptoms [7-9].

Reported histology has varied from the presence of an anomalous muscular layer to a diminution of muscle and elastin fibres [10,11]. It is not always possible to be certain whether a venous aneurysm is truly a primary phenomenon or results from a previous but long-forgotten minor trauma. The variation in pathological diagnosis may be representative of a difference in aetiology.

The complications of venous aneurysms include embolism, thrombosis and rupture [12-14]. Venous aneurysms of the portal system can be associated with portal hypertension and gastrointestinal bleeding [6,12].

Duplex scanning, Computed Tomography, Magnetic Resonance Imaging (MRI) and venography are important diagnostic modalities [15]. Isotopic ^99m^Tc human serum albumin angiography has also been used in certain circumstances [6]. Krinsky et al. describe the 'layered gadolinium sign' on MRI scanning [16]. Duplex scanning remains the first investigation of choice for most of the upper limb venous aneurysms.

The treatment of venous aneurysms depends on the site and associated symptomatology. Many symptom-free, superficial, small fusiform aneurysms without thrombus are deemed to carry a low risk of complications and therefore may remain under close surveillance with duplex scanning. Symptomatic, enlarging, popliteal and saccular aneurysms of any size or those with large fusiform aneurysms should undergo surgery [17,18]. There is no agreed size criterion that defines an aneurysm 'small' or 'large'. However, Sessa et al. had stated 20 mm as a cut-off limit for repair of popliteal venous aneurysms [15].

Surgical options for repairing venous aneurysms include: (i) excision; (ii) aneurysmorrhaphy; (iii) resection with end-to-end anastomosis and (iv) resection with interposition graft [2]. In the absence of a randomised control trial it is difficult to assess efficacy of one procedure over another. The rarity of the condition would almost preclude such a trial. There is a high incidence of postoperative venous thrombosis, particularly in popliteal venous aneurysm resection [19]. Anticoagulation should be considered to prevent post-operative venous thrombosis in selected cases. In the case described, the patient had undergone excision of the aneurysm and therefore did not merit any anticoagulation. Venous pseudoaneurysm had been successfully treated by radiological intervention (e.g., coil embolisation) [1]. Surgical excision was performed in our case because she experienced pain in the arm. The pain resolved completely after surgery.

An unrestricted search of the Medline database (from 1950 to February 2007) was performed by using the keywords 'peripheral', 'venous' and 'aneurysm'. A total of 172 articles were cited. However, only one article reported a case of venous pseudoaneurysm following venupuncture, where the patient was on long term anticoagulation [1]. While arterial pseudoaneurysms are encountered more commonly in anticoagulated patients, there is no documented relationship between venous aneurysm formation and state of coagulation profile [20]. Given this background, the present case, to our knowledge, is the first report of a true venous aneurysm formation following intravenous cannulation. This makes our case report unique.

Usually, verbal consent is obtained while establishing a peripheral cannula. According to General Medical Council, patients must be given sufficient information...to enable them to exercise their right to make informed decisions about their care [21]. This raises the question whether venous aneurysm, a rare but potentially serious complication, should be mentioned while obtaining consent for intravenous cannulation. Herein lays the importance of publishing such rare complications, as more reporting of similar cases may help provide an answer.

## Conclusion

Aneurysms of the venous system can be potentially dangerous in view of possible complications. A symptomatic acquired aneurysm of the antecubital vein secondary to intravenous cannulation has been described that was excised without any complications. Further considerations are warranted to clarify the optimum management of this rare condition and address the issue of consent.

## Abbreviations

CT – Computed tomography

MRI – Magnetic resonance imaging

GMC – General Medical Council

## Competing interests

The author(s) declare that they have no competing interests.

## Authors' contributions

DD: Performed the surgery, designed the case report, collated the information, searched literature and drafted the manuscript.

SW: Assisted with the surgery, involved in all investigations, assisted in providing a critical appraisal and review of the manuscript.

EM: Made the histological diagnoses and prepared the pathology images, advised on the format and design and assisted in providing a critical appraisal of the manuscript.

FM: Supervised and assisted with the surgery, involved in all investigations, assisted in literature search, writing and editing of the manuscript.

All authors have reviewed and approved the final manuscript.

## Pre-publication history

The pre-publication history for this paper can be accessed here:


